# The correlation between Google trends and salmonellosis

**DOI:** 10.1186/s12889-021-11615-w

**Published:** 2021-08-21

**Authors:** Ming-Yang Wang, Nai-jun Tang

**Affiliations:** 1 Department of Occupational and Environmental Health, School of Public Health, Tianjin Medical University, Tianjin, 300070 China; 2 Tianjin Key Laboratory of Environment, Nutrition, and Public Health, Tianjin Medical University, Tianjin, 300070 China; 3 Beijing Tongren Eye Center, Beijing Ophthalmology& Visual Sciences Key Laboratory, Beijing Tongren Hospital Affiliated to Capital University of Medical Sciences, Beijing, 100730 China

**Keywords:** Google trends, *salmonella*, Correlation, Predictor, Salmonellosis

## Abstract

**Background:**

*Salmonella* infection (salmonellosis) is a common infectious disease leading to gastroenteritis, dehydration, uveitis, etc. Internet search is a new method to monitor the outbreak of infectious disease. An internet-based surveillance system using internet data is logistically advantageous and economical to show term-related diseases. In this study, we tried to determine the relationship between salmonellosis and Google Trends in the USA from January 2004 to December 2017.

**Methods:**

We downloaded the reported salmonellosis in the USA from the National Outbreak Reporting System (NORS) from January 2004 to December 2017. Additionally, we downloaded the Google search terms related to salmonellosis from Google Trends in the same period. Cross-correlation analysis and multiple regression analysis were conducted.

**Results:**

The results showed that 6 Google Trends search terms appeared earlier than reported salmonellosis, 26 Google Trends search terms coincided with salmonellosis, and 16 Google Trends search terms appeared after salmonellosis were reported. When the search terms preceded outbreaks, “foods” (t = 2.927, *P* = 0.004) was a predictor of salmonellosis. When the search terms coincided with outbreaks, “hotel” (t = 1.854, *P* = 0.066), “poor sanitation” (t = 2.895, *P* = 0.004), “blueberries” (t = 2.441, *P* = 0.016), and “hypovolemic shock” (t = 2.001, *P* = 0.047) were predictors of salmonellosis. When the search terms appeared after outbreaks, “ice cream” (t = 3.077, *P* = 0.002) was the predictor of salmonellosis. Finally, we identified the most important indicators of Google Trends search terms, including “hotel” (t = 1.854, *P* = 0.066), “poor sanitation” (t = 2.895, *P* = 0.004), “blueberries” (t = 2.441, *P* = 0.016), and “hypovolemic shock” (t = 2.001, *P* = 0.047). In the future, the increased search activities of these terms might indicate the salmonellosis.

**Conclusion:**

We evaluated the related Google Trends search terms with salmonellosis and identified the most important predictors of salmonellosis outbreak.

**Supplementary Information:**

The online version contains supplementary material available at 10.1186/s12889-021-11615-w.

## Introduction

*Salmonella* infection (salmonellosis) is a common infectious disease caused by *Salmonella* bacteria. *Salmonella* bacteria belong to gram-negative bacteria in the *Enterobacteriaceae* family [1]. This kind of bacteria lives in the intestines and is transmitted by the faecal-oral transmission route [2]. The foods that can be contaminated with *Salmonella* include raw meat, poultry, seafood, raw eggs, fruits, and vegetables [3]. The symptoms of *Salmonella* infection are classified as gastroenteritis, including nausea, abdominal pain, diarrhoea, vomiting, headache, fever, and blood in stool [4]. Typhoid fever is a serious intestinal infection caused by *S. typhi* [5]. The complications of *S. typhi* infection include dehydration, bacteremia, reactive arthritis, etc. A total of 2269 outbreaks and 48,178 salmonellosis were reported in the USA from 2009 to 2018. Therefore, it is important to prevent *Salmonella* transmission with appropriate methods, such as washing hands after using toilets, and avoiding eating raw eggs. Because the sources of *Salmonella* infection are everywhere, the transmission route (faecal-oral transmission) is difficult to break. In addition, healthy people are generally susceptible to this infection. Therefore, there would be many infected people worldwide.

Internet search is a new method to monitor the outbreak of infectious diseases [6]. The internet-based surveillance system using internet data is logistically advantageous and economical to identify term-related diseases. Traditional systems to monitor infectious diseases depend on laboratory tests, physician diagnoses, and data collection by the authorities. Thus, the reporting on the emerging outbreak of infectious diseases is often delayed by one to 2 weeks in the traditional surveillance system. In the last two decades, the internet has become an important medium for the general public, public health practitioners, and doctors to obtain health-related information [7]. Internet-based surveillance systems could forecast the outbreak of infectious diseases by following online web-based activities. Therefore, it seems to be a promising strategy to monitor disease outbreaks based on internet search behaviour. Valdivia et al. used Google Flu Trends to monitor the activity of influenza in Europe [8]. They found a relationship between the disease activity peak and flu-related internet search. Seifter et al. used Google Trends to predict the epidemiology of Lyme disease [9]. Some Google Trends terms such as “tick bite” and “cough” were considered indicators of Lyme disease epidemiology. Ayyoubzadeh et al. used Google Trends to evaluate the COVID-19 incidence in Iran [10]. The researchers concerned the first wave of COVID-19 in Iran. They found that Google search terms, including handwashing, hand sanitizer, and antiseptic topics, were related to COVID-19 prevalence in Iran. The associations between COVID-19 cases and the search mentioned above terms were related to media discourse on non-pharmaceuticals interventions to mitigate the pandemic. Internet-based surveillance systems could be a useful method to evaluate outbreaks of infectious disease. Google Trends analyses the popularity of top search queries in Google Search.

Previous studies have not identified the relationship between salmonellosis and Internet searches. In this study, we examined the relationship between salmonellosis and Google Trends in the USA from January 2004 to December 2017. We hoped to reveal the correlation between Internet search trend and salmonellosis. In addition, we explored the most important Google search terms that indicate salmonellosis with multiple regression analysis. We wish to reveal the predictors of salmonellosis. Furthermore, we discovered the relationship between Google search terms with *Shigella* and *E. coli* infection to validate the function of Google Trends. We prospected Internet search trend could provide a useful strategy to monitor the infectious disease outbreak.

## Methods

### The related Google search terms

**Fig. 1:**
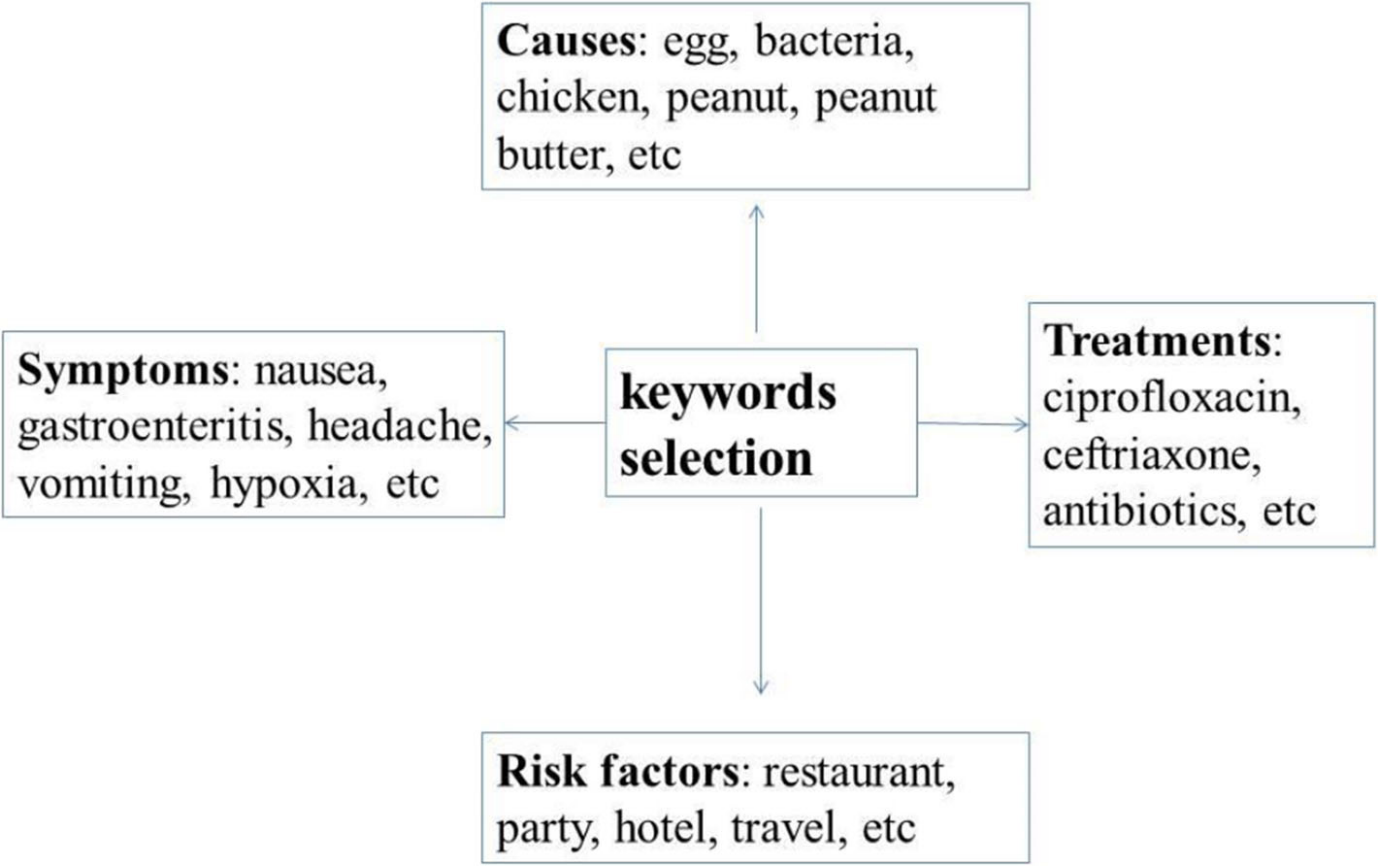
Flowchart for Google search terms selection

We determined the Google search terms related to *Salmonella* as follows. The search terms related to *Salmonella* belonged to several categories. The causes of *Salmonella* infection were infection with foods contaminated with faeces. The related terms linked to possible causes included egg, bacteria, chicken, peanut, peanut butter, butter, cookie dough, cucumber, chicken nuggets, etc. The risk factors for *Salmonella* infection include social activities to increase the possibility of infection. The related terms of social activities included restaurant, party, hotel, travel, epidemiology, etc. The symptoms of *Salmonella* infections were considered gastroenteritis. The related terms relevant to symptoms included nausea, stomach flu, gastroenteritis, headache, vomiting, hypoxia, stomach cramps, etc. *Salmonella* infection might have the same symptoms as other infectious diseases, such as *Shigella* and *E. coli*. The related terms included influenza, *E. coli* symptoms, cholera, Vibrio, etc. Because this is a bacterial infection, antibiotics are useful as a treatment for *Salmonella* infection. The related terms included ciprofloxacin, ceftriaxone, antibiotics, etc. The flowchart for Google search terms selection was shown in Fig. 1.

In addition, the total search terms in our study are listed in Supplementary Table 1.

### Google trends exploration

Google Trends is a useful tool provided by Google to analyse the search query popularity in Google. It is a public website (https://trends.google.com/trends) belonging to Google Inc. Google Trends provides keyword-related data including search volume index and geographical information about search engine users. It can be used for comparative keyword research and to discover event-triggered spikes in keyword search volume. Google Trends also allows the user to compare the relative search volume of searches between two or more terms. The values in Google Trends ranges from 0 to 100, representing search interest in different regions and times. A value of 0 indicates that the search queries are not popular enough for this search term. A value of 50 indicates that the search term is half as popular. A value of 100 indicates that the search term has peak popularity. In this study, we defined the region as “United States”, category as “Health”, and custom time range as “1/1/2004–12/31/2017” on the Google Trends website.

### The salmonellosis data collection

Salmonellosis data of US and states were obtained from the National Outbreak Reporting System (NORS) in the Centers for Disease Control and Prevention (CDC). NORS is a web-based tool to report infectious disease incidences in the United States launched by CDC [11]. NORS included enteric disease outbreaks and non-enteric disease induced by viral, bacterial, parasitic, toxin, chemical, etc. NORS also included non-enteric disease with foodborne and waterborne. It is used by territorial, state, and local health departments in US to report all waterborne and foodborne disease outbreaks and enteric disease outbreaks transmitted by contact with environmental sources, infected persons or animals, or unknown modes of transmission to CDC. We downloaded the data on salmonellosis from NORS. Because NORS has not updated the information on infectious disease outbreaks and illnesses after 2018, we defined the time range from January 2004 to December 2017. The source of the salmonella outbreaks data was downloaded from National Outbreak Reporting System (NORS) dashboard (https://wwwn.cdc.gov/norsdashboard/). The data of *Shigella* and *E. coli* were obtained from NORS, either.

### Cross correlation analysis

In this study, we used cross-correlation analysis in the SPSS software (23.0) to measure the similarity between salmonellosis and search terms in Google Trends. A cross-correlation function is used to discover the relationship between two time series variables. One time series variable value might have preceded or followed movement to another time series variable value. Cross-correlation analysis could distinguish whether movement in one time series variable values tends precedes or follows movement in the other time series variable values. With cross-correlation analysis, we tried to determine whether Google search terms were preceding or following salmonellosis.

### Multiple regression analysis

After performing cross-correlation analysis, we conducted multiple regression analysis to discover the predictor variables based on other variables. Multiple regression analysis is an extension of simple linear regression. In this study, we evaluated the significant predictor variables with salmonella infection illness. The predictor in our study was the significant predictor variables (*P* < 0.05) with salmonella infection illness in multiple regression analysis. It also provides the model overall fit and relative contribution of the predictor to the total variance explained. In this study, we combined the correlated search terms together after conducting cross-correlation analysis. We found that the predictor variables depend on the correlated search terms. In addition, we used the scikit-learn (sklearn) package in Python to conduct the machine learning. The data were divided into training dataset (2004–2016) and test dataset (2017). Data from 2004 to 2016 was used to predict salmonella outbreaks in 2017 with sklearn package.

## Results

### Characteristics of salmonellosis in the USA

**Fig. 2:**
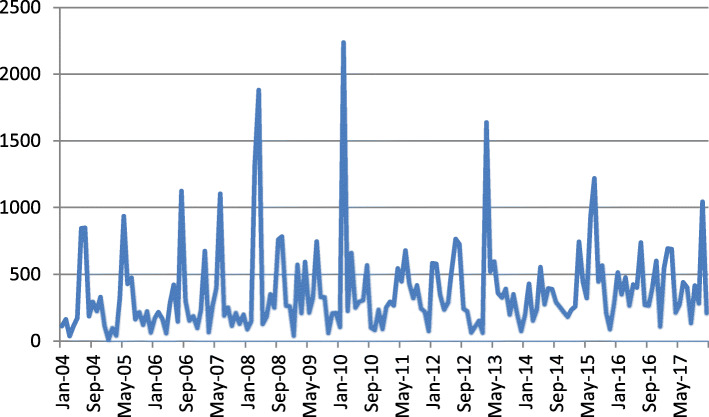
Monthly number of salmonellosis from January 2004 to December 2017 in the USA

In this study, we downloaded data on salmonellosis from NORS in the CDC from January 2004 to December 2017. A total of 2636 salmonellosis and 62,447 salmonellosis were recorded in NORS from January 2004 to December 2017. Of these reported salmonellosis, 8730 were hospitalised, and 101 died. Using these data, we calculated the salmonellosis every month during the period. The monthly change of salmonellosis was shown in Fig. 2.

### Google trends search terms that preceded salmonellosis

After conducting cross-correlation analysis, we found that 6 Google Trends search terms showed high activities prior to salmonellosis. The 6 Google search terms included salmon, nontyphoidal, foods, beef, vegetable, and ground beef (Table 1). Except for the term nontyphoidal, the other 5 terms belonged to food-related search terms. This reflected that contaminated foods could transmit *Salmonella* bacteria to healthy people. The possible contaminated foods included sea food such as salmon, meat such as beef, and vegetables.

**Table 1:** Google Trends search terms preceded salmonellosis

Google search terms	−3 months	− 2 months	−1 month	0 month	1 month	2 months	3 months
salmon	0.049	0.098	**0.215**	0.186	0.116	0.071	0.028
nontyphoidal	−0.045	0.121	**0.367**	0.130	−0.030	0.068	0.005
foods	0.082	0.121	**0.230**	0.222	0.131	0.043	0.001
beef	0.105	0.122	**0.178**	0.161	0.090	0.022	0.007
vegetable	0.070	0.131	**0.207**	0.184	0.114	−0.010	−0.031
ground beef	0.082	0.118	**0.188**	0.184	0.071	0.089	0.032

Note: The month that showed highest correlation is labelled in bold

Then, we used multiple regression analysis in these 6 search terms that preceded *Salmonella* outbreaks to identify the predictor. The results of multiple regression analysis showed that “food” (t = 2.927, *P* = 0.004) was a predictor of *Salmonella* outbreak.

### Google trends search terms that coincided with salmonellosis

In this study, we found that many Google Trends search terms coincided with salmonellosis. The related search terms included chicken, cucumber, restaurant, hotel, pork, bathroom, water, hypovolemic shock, dehydration, feel chilly, toxins, poor sanitation, bar, bradycardia, transmission, fruits, drinking water, avocado, salad, sandwich, sushi, tuna, cheese, blueberries, coleslaw, and mango (Table 2). These terms belonged to several aspects related to *Salmonella*. First, *Salmonella* can be transmitted by contaminated water and a variety of foods. The related terms included chicken, cucumber, pork, water, drinking water, fruits, avocado, salad, sandwich, sushi, tuna, cheese, blueberries, coleslaw, and mango. Second, *Salmonella* might spread from people to people. The related terms included restaurant, hotel, bathroom, poor sanitation, bar, transmission. The possible symptoms of *Salmonella* included feel chilly, bradycardia, dehydration, and hypovolemic shock.

**Table 2:** Google Trends search terms that coincided with salmonellosis outbreak

Google search terms	−3 months	−2 months	−1 month	0 month	1 month	2 months	3 months
chicken	0.063	0.115	0.173	**0.174**	0.131	0.084	0.054
cucumber	−0.020	0.042	0.136	**0.209**	0.208	0.176	0.132
restaurant	0.082	0.149	0.217	**0.222**	0.178	0.063	0.063
hotel	−0.078	0.013	0.221	**0.297**	0.181	0.085	0.114
pork	0.100	0.125	0.165	**0.194**	0.131	0.103	0.031
bathroom	0.101	0.105	0.158	**0.180**	0.138	0.092	0.087
water	0.054	0.100	0.167	**0.200**	0.172	0.104	0.075
hypovolemic shock	0.099	0.077	0.073	**0.176**	0.054	0.011	−0.040
dehydration	−0.026	0.070	0.178	**0.215**	0.158	0.104	0.079
feel chilly	0.069	−0.014	− 0.098	**0.165**	0.078	−0.052	− 0.033
toxins	0.120	0.115	0.065	**0.170**	0.072	−0.057	−0.137
poor sanitation	−0.028	0.066	0.044	**0.208**	0.082	−0.017	−0.078
bar	0.065	0.064	0.144	**0.168**	0.137	0.072	0.044
bradycardia	−0.052	−0.057	0.055	**0.166**	0.096	0.147	0.092
transmission	0.034	0.069	0.121	**0.162**	0.151	0.098	0.053
fruits	−0.009	0.077	0.233	**0.245**	0.139	0.091	0.040
drinking water	0.065	0.099	0.156	**0.169**	0.098	0.050	0.021
avocado	0.052	0.113	0.180	**0.183**	0.147	0.085	0.056
salad	0.059	0.117	0.213	**0.229**	0.194	0.116	0.076
sandwich	0.091	0.118	0.188	**0.196**	0.132	0.117	0.065
sushi	0.092	0.183	0.190	**0.223**	0.144	0.095	0.033
tuna	0.066	0.158	0.191	**0.233**	0.180	0.129	0.003
cheese	0.094	0.119	0.139	**0.158**	0.128	0.073	0.044
blueberries	−0.081	0.072	0.214	**0.275**	0.222	0.135	0.115
coleslaw	0.003	0.085	0.145	**0.191**	0.170	0.151	0.126
mango	0.024	0.129	0.201	**0.231**	0.222	0.173	0.109

Note: The month that showed highest correlation is labelled in bold

We also used multiple regression analysis in these 26 Google Trends search terms coinciding with salmonellosis to identify the predictor. The results of multiple regression analysis showed that “hotel” (t = 1.854, *P* = 0.066), “poor sanitation” (t = 2.895, *P* = 0.004), “blueberries” (t = 2.441, *P* = 0.016), and “hypovolemic shock” (t = 2.001, *P* = 0.047) were predictors of salmonellosis.

### Google trends search terms that followed after salmonellosis

We also discovered Google Trends search terms that followed the outbreaks of salmonellosis. The related search terms included *Salmonella*, poison, tomato, tomatoes, party, abdominal cramps, *Salmonella* symptoms, rash, melon, sea food, lettuce, ice cream, bbq, carneasada, watermelon, and chicken salad (Table 3). The possible contaminated foods included tomato, tomatoes, melon, sea food, lettuce, ice cream, bbq, carneasada, watermelon, and chick salad. The symptoms of *Salmonella* included *Salmonella* symptoms, abdominal cramps, and rash.

**Table 3:** Google Trends search terms that followed the outbreaks of salmonellosis

Google search terms	−3 months	−2 months	−1 month	0 month	1 month	2 months	3 months
*salmonella*	0.085	−0.048	− 0.059	− 0.009	− 0.090	0.173	**0.251**
poison	−0.176	− 0.044	0.118	0.200	**0.247**	0.239	0.176
tomato	−0.035	− 0.018	0.098	0.128	0.142	**0.399**	0.291
tomatoes	−0.004	0.044	0.098	0.140	0.185	**0.204**	0.194
party	0.022	−0.066	0.014	0.044	0.126	**0.177**	0.124
abdominal cramps	−0.059	0.020	0.081	0.175	0.108	0.064	**0.182**
*salmonella* symptoms	0.070	−0.034	−0.039	0.024	−0.052	0.134	**0.235**
rash	−0.005	0.062	0.142	0.183	**0.184**	0.149	0.135
melon	−0.035	−0.046	0.121	0.211	**0.214**	0.168	0.157
sea food	0.041	0.093	0.125	0.190	**0.238**	0.120	0.019
lettuce	0.020	0.004	0.064	0.066	0.097	0.110	**0.208**
ice cream	0.002	0.108	0.177	0.232	**0.233**	0.149	0.075
bbq	0.029	0.089	0.150	0.160	**0.188**	0.146	0.130
carneasada	0.019	0.067	0.147	0.164	**0.184**	0.136	0.124
watermelon	−0.097	0.017	0.125	0.199	**0.203**	0.185	0.181
chicken salad	−0.018	0.084	0.195	0.225	**0.227**	0.175	0.132

Note: The month that showed highest correlation is labelled in bold

We also used multiple regression analysis in these 16 Google Trends search terms that followed the outbreaks of salmonellosis to identify the predictor. The results of multiple regression analysis showed that “ice cream” (t = 3.077, *P* = 0.002) was a predictor of *Salmonella* outbreak.

### Correlation between Google trends search terms and salmonellosis in Massachusetts and California

We tried to use a smaller geographical unit (state) to explore the relation. Massachusetts and California were selected to analyze. Massachusetts is one of the states to have highest population density in USA. California has most cases of salmonellosis from 2004 to 2017. So, we selected Massachusetts and California to explore the relation between salmonellosis and Google Trends. The results of Massachusetts and California were shown in Tables 4 and 5.

**Table 4:** The Google Trends search terms related with salmonellosis in Massachusetts

Google search terms	−3 months	−2 months	−1 month	0 month	1 month	2 months	3 months
foods	**0.166**	0.087	0.102	0.075	0.140	0.089	0.130
chicken	0.126	0.098	0.113	0.057	0.105	**0.232**	0.136
water	0.123	0.152	0.106	0.106	0.070	**0.189**	0.162
dehydration	0.063	0.084	−0.017	0.186	0.018	**0.223**	0.063
bar	0.088	**0.189**	−0.015	0.038	0.005	0.103	0.097
fruits	0.037	**0.152**	0.058	0.073	0.018	−0.003	0.127
cheese	0.149	0.095	0.116	0.115	0.119	**0.160**	0.139
blueberries	0.116	0.037	−0.117	−0.061	0.025	**0.224**	0.064
abdominal cramps	0.006	0.060	−0.015	**0.167**	0.050	0.015	0.137
rash	0.039	0.050	0.087	0.106	0.131	**0.193**	0.127
seafood	0.099	0.125	**0.246**	0.071	0.053	0.144	−0.062
lettuce	−0.070	0.052	−0.043	− 0.033	0.000	**0.172**	0.017
icecream	0.028	0.042	0.021	0.020	0.065	**0.196**	0.086
bbq	−0.007	−0.021	0.100	0.045	0.096	**0.154**	0.058
outbreak	0.199	0.244	**0.434**	0.017	0.021	0.086	0.091
avocado	0.129	**0.209**	0.181	0.101	0.185	0.146	0.128

Note: The month that showed highest correlation is labelled in bold

**Table 5:** The Google Trends search terms related with salmonellosis in California

Google search terms	−3 months	−2 months	−1 month	0 month	1 month	2 months	3 months
transmission	0.019	−0.001	0.077	0.027	0.016	0.084	**0.156**
salmonella	0.118	−0.046	− 0.041	− 0.003	−0.004	− 0.043	**0.376**
salmonella symptoms	−0.033	− 0.102	− 0.108	− 0.094	− 0.005	−0.061	**0.252**
carneasada	−0.026	−0.003	− 0.033	**0.211**	− 0.025	−0.025	− 0.033

Note: The month that showed highest correlation is labelled in bold

In Massachusetts, we found that 6 Google Trends search terms showed prior to salmonellosis. The Google Trends search terms included foods, bar, fruits, seafood, avocado, outbreak. In addition, search term “abdominal cramps” was coincided with salmonellosis. 9 Google Trends search terms were followed after salmonellosis including dehydration, rash, lettuce, etc. In California, 4 Google search terms were related with salmonellosis including transmission, salmonella symptoms, uveitis, etc.

### Correlation between Google trends search terms with *Shigella* and *E. coli* in US

Furthermore, we analyzed the similar infectious diseases including *Shigella* and *E. coli* in US. We found that several Google Trends search terms were with *Shigella* and *E. coli*. The results were shown in Tables 6 and 7.

**Table 6:** The Google Trends search terms related with *E. coli*

Google search terms	− 3 months	−2 months	− 1 month	0 month	1 month	2 months	3 months
poison	0.291	0.415	**0.445**	0.355	0.144	−0.056	− 0.204
tomato	0.202	**0.458**	0.327	0.221	0.114	−0.085	−0.145
chicken	0.209	**0.218**	0.150	0.119	0.106	0.025	0.004
contamination	0.011	0.183	0.006	−0.085	**0.227**	0.101	0.081
restaurant	0.239	**0.286**	0.165	0.154	0.098	−0.005	− 0.043
hotel	0.230	0.297	**0.298**	0.203	−0.033	− 0.150	− 0.274
cookie dough	0.133	0.081	**0.197**	0.059	0.097	0.030	0.045
dehydration	0.252	0.224	**0.311**	0.299	0.150	0.014	−0.051
abdominal cramps	0.122	0.195	0.173	**0.263**	0.251	0.082	−0.092
stomach cramps	0.144	0.166	0.160	**0.172**	0.165	0.114	0.048
contaminated food	−0.034	**0.329**	−0.064	0.006	−0.017	0.070	0.003
transmission	0.225	**0.236**	0.205	0.173	0.113	0.068	0.016
rash	0.227	0.291	**0.300**	0.264	0.196	0.085	−0.010
weakness	0.138	0.165	**0.229**	0.219	0.165	0.137	0.019
headache	0.129	0.137	0.147	**0.166**	0.176	0.135	0.090
bar	**0.268**	0.248	0.201	0.140	0.079	0.023	−0.015
Ecoli symptoms	− 0.016	0.083	0.084	0.052	**0.219**	0.097	0.018
flood	0.117	**0.416**	0.204	0.095	0.126	0.040	−0.053
melon	0.197	**0.318**	0.311	0.280	0.190	0.121	−0.077
lettuce	0.102	0.179	**0.189**	0.074	0.024	0.063	0.045
chicken salad	0.260	**0.341**	0.316	0.245	0.149	0.014	−0.088
smoked chicken	0.209	0.246	**0.275**	0.225	0.177	0.111	0.029
coleslaw	0.216	0.229	**0.243**	0.203	0.107	0.015	−0.051
iceberg lettuce	0.181	**0.212**	0.195	0.145	0.103	0.041	−0.014
carneasada	0.265	0.281	**0.247**	0.203	0.163	0.081	−0.018
bbq	0.254	**0.271**	0.244	0.237	0.123	0.077	−0.004
sushi	0.238	**0.284**	0.196	0.128	0.023	−0.078	− 0.055
fruits	0.179	**0.243**	0.156	0.120	0.059	−0.097	−0.113
salad	0.273	**0.309**	0.228	0.155	0.079	−0.072	−0.069

**Table 7:** The Google Trends search terms related with *Shigella*

Google search terms	−3 months	−2 months	−1 month	0 month	1 month	2 months	3 months
diarrhea	0.420	0.433	**0.417**	0.416	0.416	0.403	0.407
food poisoning	**0.389**	0.347	0.297	0.242	0.250	0.315	0.344
bacteria	0.046	−0.006	0.027	0.130	**0.163**	0.033	−0.010
egg	0.282	0.297	0.265	0.312	**0.298**	0.257	0.262
chicken	0.343	**0.413**	0.383	0.403	0.387	0.298	0.377
Shigella	0.006	0.066	0.052	**0.315**	0.208	0.038	0.058
cookie dough	0.315	0.313	**0.362**	0.307	0.324	0.311	0.307
restaurant	0.247	**0.342**	0.302	0.234	0.245	0.243	0.230
party	0.296	0.288	0.309	**0.492**	0.418	0.370	0.358
antibiotics	0.373	0.376	0.370	0.373	0.393	0.389	**0.398**
sprouts	0.374	0.411	**0.359**	0.368	0.370	0.352	0.379
hypovolemic shock	0.276	0.199	0.171	**0.349**	0.295	0.127	0.166
hypoxia	0.210	0.165	0.256	**0.344**	0.306	0.210	0.241
stomach cramps	0.380	0.407	**0.408**	0.397	0.389	0.383	0.393
abdominal cramps	0.207	0.282	0.267	**0.346**	0.296	0.260	0.257
nausea	0.398	0.403	0.387	0.396	0.406	0.399	**0.408**
headache	0.352	0.376	0.373	**0.389**	0.398	0.380	0.388
feel chilly	−0.004	0.048	0.070	0.078	**0.182**	0.078	0.124
shock	0.206	0.220	0.210	**0.318**	0.282	0.173	0.233
infection	0.341	0.350	0.337	0.348	**0.377**	0.317	0.348
weakness	0.347	0.367	0.397	0.416	**0.418**	0.367	0.376
peritonitis	0.090	0.162	0.123	0.170	**0.212**	0.068	0.145
ceftriaxone	0.408	0.393	0.400	0.425	**0.449**	0.380	0.358
vaccines	0.254	0.209	0.239	**0.293**	0.298	0.203	0.203
malaise	0.305	0.315	0.366	**0.405**	0.402	0.352	0.350
raw eggs	0.401	**0.409**	0.353	0.355	0.373	0.365	0.384
cow milk	0.187	**0.222**	0.146	0.143	0.118	0.086	0.200
red meat	0.339	0.314	0.242	**0.429**	0.360	0.253	0.269

### Identifying the most important Google search terms that indicate salmonellosis outbreak

**Fig. 3:**
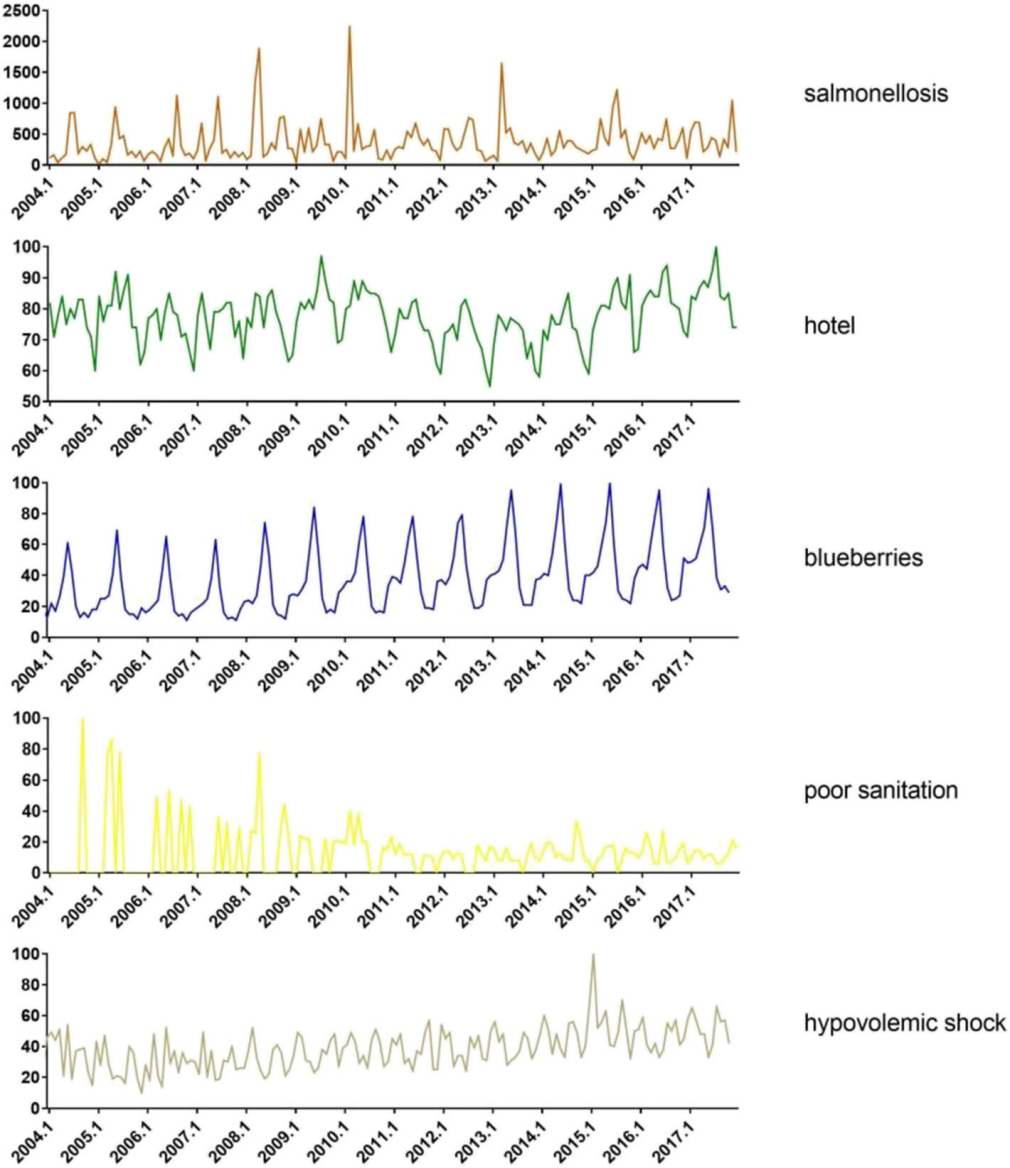
The trends of salmonellosis and best-suited search terms

Finally, we tried to identify the most important Google search terms that correlated with salmonellosis. Multiple regression analysis was conducted. We combined the Google search terms from the previous three sections that preceded, coincided and followed salmonellosis. A total of 48 Google search terms were correlated with salmonellosis in cross-correlation analysis. Through multiple regression analysis, 4 search terms were identified as the most important indicators of salmonellosis outbreaks. These 4 search terms were “hotel” (t = 1.854, *P* = 0.066), “poor sanitation” (t = 2.895, *P* = 0.004), “blueberries” (t = 2.441, *P* = 0.016), and “hypovolemic shock” (t = 2.001, *P* = 0.047). Interestingly, the results in this section for a total of 48 Google search terms were almost the same as those for 26 Google Trends search terms that coincided with salmonellosis. These 4 most important indicators referred to different aspects of *Salmonella* infection. The time trends including salmonellosis and best-suited search terms were showed in Fig. 3.

Moreover, we also tried to processed data from 2004 to 2016 to predict salmonella outbreaks in 2017. We used the scikit-learn (sklearn) software in Python to conduct the machine learning. The data were divided into training dataset (2004–2016) and test dataset (2017). We used 4 best-suited search terms (hotel, hypovolemic shock, poor sanitation, blueberries) to conduct the machine learning. The result was shown in Supplementary Fig. 1.

## Discussion

*Salmonella* is a common food borne pathogen in the USA [12]. Foods are the source of most of *Salmonella* infections. Contaminated foods and water that look and smell untainted can infect healthy people [13]. In addition, *Salmonella* can be transmitted from pets to people and from people to people [14, 15]. Most *Salmonella* patients suffer from fever, diarrhoea, stomach cramps, nausea, vomiting, and headache [16]. Infections can be serious for infants and older adults [17, 18]. Because *Salmonella* infection is foodborne, it is also preventable. First, people should wash hands before and after preparing foods [19]. Second, different kinds of foods, such as raw meat, eggs, and seafood, should be separated.

In our study, we discovered the correlation between salmonellosis with Google search terms. We found that a total of 48 Google search terms were associated with salmonellosis. These terms represented different aspects of salmonellosis. The first group of Google search terms are associated with foods and fruits. The search terms in this category included foods, salmon, beef, vegetable, ground beef, chicken, cucumber, pork, fruits, avocado, salad, sandwich, sushi, tuna, cheese, blueberries, coleslaw, mango, tomato, tomatoes, melon, sea food, lettuce, ice cream, carneasada, watermelon, and chicken salad. A variety of foods and fruits were associated with salmonellosis outbreaks. This result indicated that *Salmonella* was very common in foods and fruits. Foods and fruits are considered one of most important sources of salmonellosis outbreaks [20]. The results of multiple regression analysis indicated that “blueberries” was a predictor of salmonellosis outbreaks. Blueberries have a relatively short shelf life compared with other kinds of fruits, such as melon and watermelon [21]. Previous studies found that *Salmonella* could grow on harvested blueberries at retail display temperatures, while *Salmonella* did not grow on strawberries under the same conditions [22]. The complicated production chain of blueberries might contribute to *Salmonella* contamination. Blueberries rely heavily on humans to harvest, which promotes Salmonella transmission. Many reported outbreaks of Salmonella were associated with blueberries [23]. Various methods have been developed to reduce *Salmonella* in blueberries. These methods include antimicrobial solution with freezing, ozone, and UV light [24, 25]. Apart from blueberries, “ice cream” was also a predictor of *Salmonella* outbreaks. Ice cream, especially homemade creams, contains raw eggs and milk. Raw eggs and milk might be contaminated with *Salmonella*. A nation-wide outbreak of salmonellosis has been reported to be transmitted from ice cream in the USA [26]. The researchers discovered that one brand of ice cream (Schwan’s) was responsible for the nation-wide outbreak of *Salmonella* infection. The FDA also detected *Salmonella* in ice cream production facilities in the US [27]. To avoid the risk of *Salmonella* infection, people should make ice cream with pasteurized egg products or pasteurized shell eggs. Other methods to make safe ice cream are to use cooked egg base or to prepare ice cream without eggs.

Apart from food-related Google search terms, we also found that salmonellosis were correlated with public places and activities that might be contaminated with *Salmonella*. Such places and activities included hotels, bathrooms, bars, parties, and bbq. In these public places and activities, healthy people might be infected from foods and facilities. For example, it was reported that sauces and salsa prepared at hotels in Dallas and Texas were considered vehicles for salmonella outbreaks. The investigation pointed out that hotel food workers infected with Salmonella were linked this salmonellosis outbreak, which affected 617 people from 46 states in the US [28]. In our study, the multiple regression analysis also showed that “hotel” and “poor sanitation” were predictors of salmonellosis. If public places such as hotels and bars have poor sanitation, people can easily be infected with *Salmonella*, leading to salmonellosis outbreaks.

Finally, we also found that “hypovolemic shock” was a predictor of salmonellosis outbreaks. Hypovolemic shock is a life-threatening symptom of *Salmonella* infection. In typhoid fever and paratyphoid fever, severe vomiting and diarrhoea cause electrolyte and liquid loss, leading to microenvironment imbalance [29]. Fluid and electrolyte imbalance was related to a decrease in arterial pressure and circulating blood volume. Hypovolemic shock and septic shock would occur in this situation. People with Salmonella infection suffer from severe vomiting and diarrhoea. They might be afraid that this situation would lead to hypovolemic shock. Therefore, the search term “hypovolemic shock” was correlated with salmonellosis.

The results of single state (Massachusetts and California) were not meets expectation very well. We inferred the data collection style of States in NORS dataset might be the reason. In NORS dataset with state, the illnesses were divided into multistate outbreaks and single-state outbreaks. Multistate outbreak might include illnesses from other states. In Massschusetts, we found that Google search terms including bar, fruits, seafood, avocado were prior to salmonellosis. These terms might be the reasons of salmonellosis outbreak in Massachusetts. Dehydration and rash were followed after salmonellosis. These terms might be the delayed symptoms of *salmonella* infection.

Lastly, we tried to predict 2017 salmonellosis outbreaks based on 2004–2016 dataset with machine learning. However, the results of prediction of future outbreak based on the specific search terms were not meets expectation very well. We inferred some reasons might explain the results. Firstly, *salmonella* was transmitted by fecal-oral transmission route. Human and animals could be infected with *salmonella* by contaminated food or water. Salmonellosis outbreaks were not regularly. Secondly, our study only included the dataset of Google Trends. Other data might influence the Salmonellosis outbreaks such as weather, health status, population density were not enrolled in our study.

Our study mainly focused on the effect of Google Trends to monitor the salmonellosis outbreaks. The purpose of Big Data utilization is now shifting toward forecasting from monitoring. So, the directions of the studies in the future should focus on accurate and precise forecasting of infectious disease outbreak.

There have some limitations in this study. Firstly, we used the Internet search engine Google Trends to evaluate the results. Other Internet engines such as Twitter, Facebook have not enrolled in our study. Secondly, we analyzed the data monthly owning to the Google Trends provided the data monthly. The seasonal data has not been evaluated in this study. In the future, the analysis could be adjusted to seasonality if the seasonal data were available. Thirdly, the data of US was analyzed in this study. Other regions worldwide such as European, Asia have not been investigated. Lastly, the prediction of salmonellosis outbreaks has not meets expectation in our study. In the future, the prediction of infectious disease based on the specific search terms could be investigated if the raw data was expanded.

## Conclusion

In this study, we discovered the correlation between Google search terms and salmonellosis in the US from 2004 to 2017. We investigated related Google terms with salmonellosis and identified most important indicators of salmonellosis outbreak. We found that Google Trend was a useful method to monitor salmonellosis outbreak. We also validated the Google Trends with *Shigella* and *E. coli*. Thus, we considered Google Search could be used to monitor infectious disease.

## Supplementary Information

**Additional file 1.Supplementary Table 1.** Total search terms in Google Trends.

**Additional file 2. Supplementary Figure 1. **The prediction of salmonella outbreaks in 2017.

## Data Availability

The data that support the findings of this study are available from the corresponding author.
