# Supplementary material for: The correlation between Google trends and salmonellosis
Source: BMC Public Health. 2021 Aug 21;21:1575. doi: 10.1186/s12889-021-11615-w (PMC8379030; doi:10.1186/s12889-021-11615-w)
Supplement: Supplementary file 1 — Additional file 1.Supplementary Table 1. Total search terms in Google Trends. [file 12889_2021_11615_MOESM1_ESM.docx]

Supplementary table 1. Total search terms in Google Trends

| Search terms |
| --- |
| Salmonella, typhoid, typhoid fever, diarrhea, poison, poisoning, Ecoli, food poisoning, disease outbreak, egg, bacteria, chicken, peanut, peanut butter, butter, salmonella enterica, product recall, salmonella typhimurium, tomato, Shigella, turtles, contamination, incubation period, cookie dough, salmon, cucumber, restaurant, party, hotel, travel, antibiotics, epidemiology, turkey, meat, pork, bathroom, dry environments, water, heating food, reptiles, amphibians, feces, gastrointestinal disease, dust, children, elder, nontyphoidal, salmonella enterica Typhimurium, Enterica, enteritidis, fever, hepatosplenomegaly, respiratory symptoms, sprouts, chicken nuggets, potpies, vaccine, birds, vomiting, diarrhea, hypovolemic shock, toxemia, hypoxia, salmonellosis, dehydration, feel chilly, food infections, stomach cramps, abdominal cramps, nausea, stomach flu, gastroenteritis, headache, blood in the stool, CDC, influenza, salmonella symptoms, enterobacteriaceae, Escherichia coli, endotoxins, shock, lipopolysaccharide, foods, contagious, infection, toxins, contaminated food, rash, weakness, abdominal pain, constipation, confusion, peritonitis, ciprofloxacin, ceftriaxone, contaminated water, washing facilities, vaccines, enteric fever, paratyphoid fever, poor sanitation, poor hygiene, bar, malaise, fever fluctuations, bradycardia, bloody nose, epistaxis, fecal oral route, bloody stools, cooking, peanut salmonella, peanut butter salmonella, symptoms of salmonella poisoning, ecoli symptoms, cholera, vibrio, giardiasis, raw chicken, raw eggs, FDA, typhoidmary, diphtheria, iritis, uveitis, transmission, salmonella patients, beef, flood, vegetable, red meat, cow milk, raw milk, green vegetable, melon, bean sprout, seafood, fruits, intestinal bleeding, drinking water, avocado, lettuce, ground beef, salad, sandwich, roasted, cantaloupe, potato, sauces, sushi, tuna, icecream, bbq, cheese, lamb, marsala, blueberries, pancit, sausage, salami, carneasada, watermelon, carnitas, coleslaw, smoked, buffet, alfalfa sprouts, smoked chicken, iceberg lettuce, chicken salad, tomatoes, mango |
