# Supplementary figures and images for: The correlation between Google trends and salmonellosis
Source: BMC Public Health. 2021 Aug 21;21:1575. doi: 10.1186/s12889-021-11615-w (PMC8379030; doi:10.1186/s12889-021-11615-w)

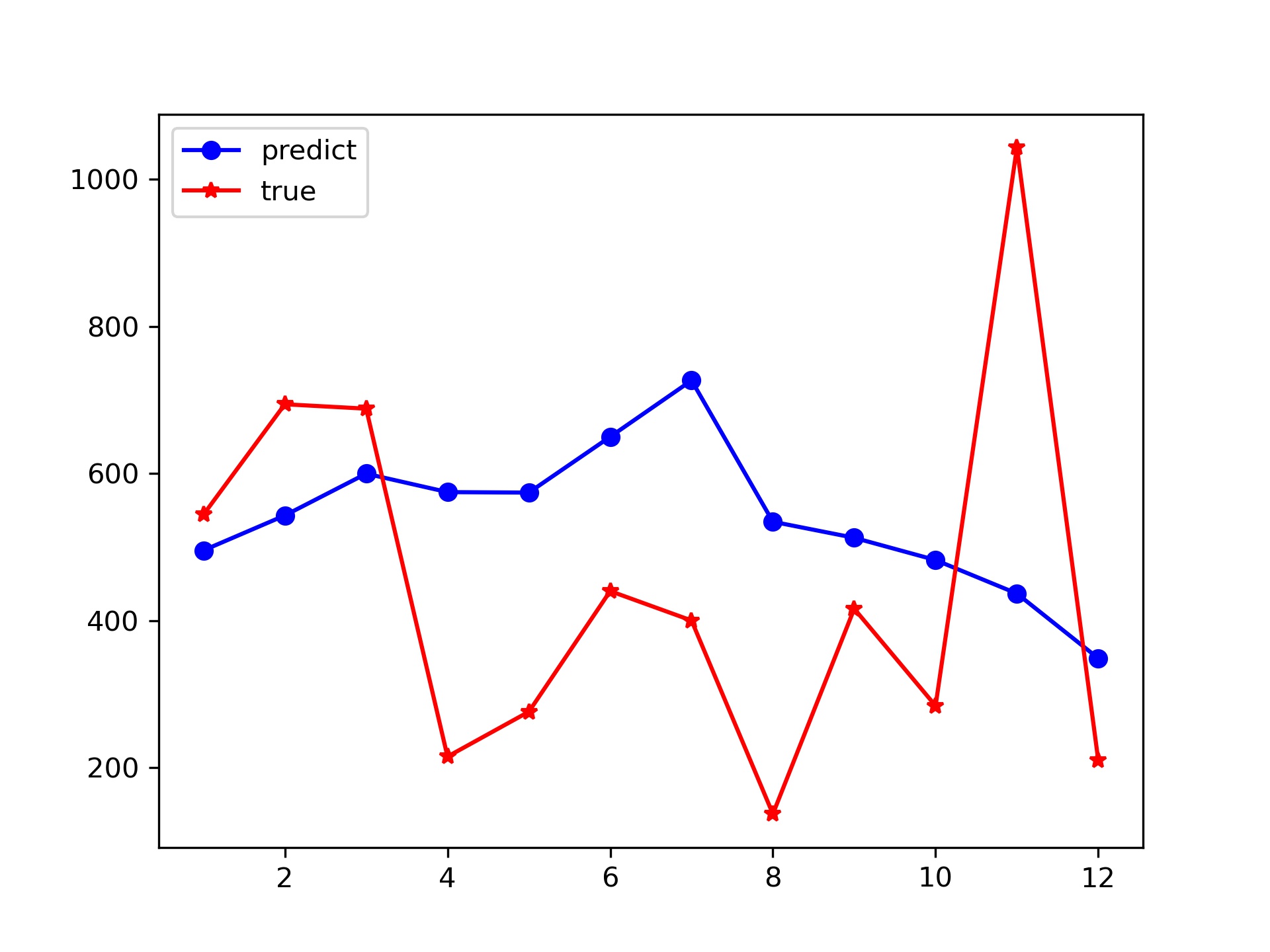

Supplement: Supplementary file 2 — Additional file 2. Supplementary Figure 1. The prediction of salmonella outbreaks in 2017. [file 12889_2021_11615_MOESM2_ESM.jpg]
